# Second Victim Trauma Recovery Pathway of Emergency Nurses After Patient Safety Incidents: A Grounded Theory Study

**DOI:** 10.1155/jonm/4175385

**Published:** 2026-04-30

**Authors:** Tao Lin, Yanzi Zhang, Yongli Gao

**Affiliations:** ^1^ Emergency Department of West China Hospital, Sichuan University/West China School of Nursing, Sichuan University, Chengdu, China, scu.edu.cn; ^2^ Institute of Disaster Medicine, Sichuan University, Chengdu, China, scu.edu.cn; ^3^ Nursing Key Laboratory of Sichuan Province, Chengdu, China, scu.edu.cn; ^4^ Department of Emergency Medicine, Laboratory of Emergency Medicine, West China Hospital, West China School of Medicine, Sichuan University, Chengdu, China, scu.edu.cn

**Keywords:** emergency nurse, grounded theory, nursing management, patient safety incident, secondary victim, trauma recovery

## Abstract

**Objective:**

Drawing on Strauss and Corbin’s grounded theory, this study systematically examined the internal mechanisms underlying trauma recovery among emergency nurses as secondary victims of patient safety incidents. A context‐specific theoretical model was developed to provide empirical evidence to inform the design of targeted support strategies for nursing managers.

**Methods:**

A combination of purposive and theoretical sampling was used to recruit 18 frontline emergency nurses who had experienced patient safety incidents in the emergency department of a tertiary Grade A hospital in Chengdu between January and June 2025. Data were collected through semistructured, in‐depth interviews. NVivo 12.0 was applied to conduct open, axial, and selective coding. Additional interviews were undertaken to confirm theoretical saturation.

**Results:**

The analysis yielded 68 initial categories, 22 subcategories, and 7 core categories. These findings informed the construction of a circular, dynamic theoretical model of trauma recovery comprising four sequential stages: trauma stress trigger, cognitive appraisal and emotional response, acquisition of intervention and support, and recovery reconstruction and career reorientation. Managerial support and proactive coping strategies emerged as key factors influencing trauma recovery.

**Conclusion:**

Trauma recovery among emergency nurses as secondary victims of patient safety incidents represents a dynamic, cyclical process shaped by the interaction of individual, organizational, and contextual factors. Nursing managers can apply the stage‐specific features of this model to establish a stratified and integrated support system, thereby improving psychological recovery, sustainable career development, emergency nursing management, and patient safety outcomes.

**Implications for Nursing Management:**

Emergency nursing managers should implement phased support strategies: immediate nonblaming crisis intervention with temporary duty adjustment during acute stress; structured psychological counseling and peer support groups during emotional processing; accessible three‐tier support framework combining managerial, professional, and organizational resources; and career development opportunities transforming trauma into professional growth. These measures operationalize “just culture” principles, fostering nurses’ well‐being, team resilience, and patient safety.

## 1. Introduction

Patient safety is a critical concern in healthcare systems worldwide, directly influencing the quality of medical care and patient protection [1, 2]. Nursing teams, as the primary clinical workforce, play an indispensable role in maintaining patient safety [1, 2]. Healthcare providers who experience unexpected medical errors or adverse events resulting in patient harm and suffer related trauma are referred to as secondary victims [1, 2]. The core characteristics of these victims include a sense of responsibility, traumatic stress response, and impaired professional functioning [3]. Second victims are fundamentally distinct from those experiencing burnout (prolonged work stress) [4], compassion fatigue (long‐term caregiving depletion) [5], and moral injury (witnessing moral transgressions) [6].

The distinctiveness of secondary victims lies in the convergence of sudden adverse events, clear responsibility attribution, and threat to professional identity [7, 8]. Existing research demonstrates that second victims frequently experience occupational burnout, reduced job satisfaction, and increased turnover intentions [9–11], threatening nursing team stability and creating a cycle of workforce shortages, deteriorating care quality, and recurrent safety incidents.

The emergency department (ED), characterized by rapidly evolving conditions, complex processes, high workloads, and frequent conflicts, is a high‐risk setting for patient safety incidents [12, 13]. The ED environment provides a distinct context for studying secondary victims because of time compression (shortened psychological buffers), role ambiguity (team‐based decision‐making), repetitive exposure (high‐frequency traumatic events), and lack of support (shift work disrupting peer continuity) [14–16]. Existing research has predominantly focused on operating rooms or medical wards [12, 17], overlooking how the ED’s high‐frequency, high‐pressure environment reshapes mechanisms of occurrence and recovery trajectories. Emergency nurses work under sustained high‐pressure conditions, which place them at heightened risk [17]. Despite their growing recognition, research focusing on these challenges remains limited and predominantly quantitative [7, 10].

Three key theoretical gaps persist: process black‐boxing (cross‐sectional designs obscuring dynamic recovery mechanisms), decontextualization (insufficient examination of specific contexts such as EDs), and theoretical fragmentation (scattered explanatory frameworks lacking integration) [12, 17]. This study aimed to bridge these gaps by constructing a dynamic recovery theory for emergency settings. Current research focuses on status investigations in specialized departments [18], with ED studies limited to psychological experiences and resourcefulness levels [17], providing limited insight into dynamic trauma recovery processes [19, 20].

In terms of theoretical contribution, this is the first study to construct a process theory of dynamic recovery for ED secondary victims, expanding trauma psychology application to acute settings. Regarding practical value, this study provides evidence‐based guidance for ED managers to design phased, precise psychological support programs, improving medical personnel well‐being and patient safety culture.

## 2. Materials and Methods

### 2.1. Study Design

This study adopted a grounded theory design following Strauss and Corbin’s methodological framework [21], adhering to pragmatic epistemology that pursues value neutrality rather than absolute objectivity. Specific measures included procedural neutrality (systematic coding procedures to minimize subjective arbitrariness), triangulation (second author independently reviewing coding schemes and challenging interpretations), theoretical saturation (ensuring data‐driven conclusions through continuous sampling and comparison), and memo documentation (detailed recording of analytical decision‐making processes for traceability).

### 2.2. Study Participants

#### 2.2.1. Sampling Strategy

A combination of purposive and theoretical sampling was used [22] to recruit 18 frontline emergency nurses who had experienced patient safety incidents in the ED of a tertiary Grade A hospital in Chengdu from January to June 2025. The initial three participants were selected through purposive sampling to ensure diversity in event types and work experience. Subsequently, theoretical sampling was guided by emerging categories. During the open coding of the fifth dataset, the category “professional identity threat” emerged, but its dimensions (temporary vs. persistent) remained unclear. Following Straussian principles, the next batch deliberately included nurses with different professional identity trajectories (e.g., newly recruited vs. senior nurses) to examine variation in conditions and attribute ranges. This process was conducted over four rounds, with the criteria adjusted after each round until new data no longer generated new attributes or relationships (theoretical saturation achieved).

Inclusion criteria were as follows: (1) valid nurse practice certificate and a minimum of one year of ED experience; (2) direct involvement in at least one patient safety incident (medication errors, falls, unplanned extubation, or delayed recognition of deterioration); (3) ability to communicate clearly and describe experiences and emotional responses; and (4) voluntary participation with written informed consent. The exclusion criteria were as follows: (1) history of mental illness or psychological disorders and (2) indirect involvement in incidents (nondirect caregiving roles).

Theoretical saturation determined the sample size, with recruitment being discontinued when no new concepts emerged. A total of 18 nurses were included (16 females and 2 males), reflecting the characteristics of China’s emergency nursing workforce.

#### 2.2.2. Participant Characteristics

Within the Straussian framework, this distribution is regarded as a contextual background that influences the selection of action strategies. The analysis focused on sex as a mediating variable, examining how it shapes coping strategies (e.g., the “resilience” expectation). Data from the two male participants were used to examine whether the categories were sensitive to sex differences. Theoretical saturation ensured that their experiential patterns were fully incorporated into the model. Participant characteristics are presented in Table 1.

**TABLE 1:** General information of study participants and distribution of adverse events experienced (*n* = 18).

Respondent ID	Sex	Years of service	Record of formal schooling	Professional ranks and titles	Types of patient safety incidents experienced
N1	Female	2	Junior college	Nurse	Incorrect medication dosage
N2	Male	11	Undergraduate course	Nurse‐in‐charge	Patient falls from the bed
N3	Female	3	Undergraduate course	Nurse	Incorrect oral medication administration
N4	Female	4	Undergraduate course	Primary nurse	Development of Stage III pressure injury
N5	Female	5	Undergraduate course	Primary nurse	Soft tissue injury due to the patient’s fall
N6	Female	12	Undergraduate course	Primary nurse	Local swelling due to intravenous infusion extravasation
N7	Female	9	Undergraduate course	Primary nurse	Specimen collection error
N8	Female	8	Undergraduate course	Primary nurse	Delayed recognition of patient condition deterioration
N9	Female	9	Undergraduate course	Primary nurse	Unplanned extubation
N10	Female	16	Undergraduate course	Primary nurse	Patient missing
N11	Female	11	Undergraduate course	Primary nurse	Exposure to body fluids
N12	Male	6	Undergraduate course	Primary nurse	Patient aspiration
N13	Female	13	Undergraduate course	Nurse in charge	Tube displacement during transport
N14	Female	3	Junior college	Nurse	Burn injury
N15	Female	18	Undergraduate course	Nurse in charge	Skin injury caused by excessive restraint
N16	Female	7	Undergraduate course	Primary nurse	Needle‐stick injury
N17	Female	12	Undergraduate course	Primary nurse	Assault by a person with mental or behavioral disorders
N18	Female	6	Master’s	Primary nurse	Delayed prehospital emergency response

### 2.3. Ethical Considerations

This study adhered to the Declaration of Helsinki. Ethical approval was obtained from the hospital’s ethics review committee (approval number: 2020 [833]). Participants were fully informed of the study objectives and procedures, data handling, and their rights to voluntary participation and to withdraw without their employment or career being impacted. Written informed consent was obtained. To ensure confidentiality, privacy, and ethical rights, interview data were anonymized using numerical identifiers, and audio recordings and transcripts were securely stored on encrypted hard drives, accessible only to the research team.

### 2.4. Data Collection

The semistructured interview guide was revised based on the literature review and prestudy interviews with two emergency nurses, covering event recall, immediate reactions, coping strategies, support needs, and long‐term impacts. The guide was dynamically adjusted as analysis progressed; for instance, a “re‐exploration of professional meaning” probe was added after the fourth round to deepen theoretical sampling during later recovery stages. The guide focused on (1) detailed descriptions of incidents, participants’ roles, and immediate consequences; (2) emotional, cognitive, and behavioral responses across stages; (3) coping strategies and support from managers, colleagues, and family; (4) changes in work attitudes, professional identity, and clinical practice during recovery; and (5) perceived needs and recommendations for managerial support.

Interviews were conducted by two postgraduate nursing students who had received training in qualitative interviewing. Both were registered nurses without ED experience, receiving 8 h of Straussian interview training, with the first two interviews observed by the first author to ensure neutral inquiry. Interviewers wrote concluding memos documenting interactions for contextual reference. They had no prior working relationship with the participants; initial contact was facilitated by the unit head nurse. The participants were informed of interviewers’ student status and that their responses would not affect performance evaluations.

Interviews occurred in a private hospital room and scheduled to avoid peak workload periods. Each lasted 40–90 min. With consent, interviews were audio‐recorded, and nonverbal cues were documented in field notes. Recordings were transcribed verbatim within 24 h. Data collection and analysis were conducted concurrently. Theoretical saturation was deemed achieved when no new categories emerged, after which recruitment was terminated.

### 2.5. Data Analysis

Data management and coding visualization were conducted using NVivo 12, with all analytical decisions based on the Straussian analysis procedure [21]. The software was used to store, retrieve, and visualize coding relationships. Code naming, category construction, and the application of the paradigm model were accomplished through inter‐researcher discussions. Analytical memos were written independently of the software to ensure depth of conceptual thinking. Interviews were conducted in Mandarin and audio‐recorded for verbatim transcription. Analysis was performed using the original Chinese text, with all coding, memos, and theoretical models developed in Chinese. For international publication, key quotations were back‐translated by bilingual researchers to ensure semantic equivalence, and the final analytical results were subjected to semantic proofreading to preserve the interpretative nuances of the Chinese context.

The data analysis was primarily led by the first author, who was responsible for initial coding and category construction; the second author independently reviewed the coding scheme and proposed revisions. Theoretical integration was completed through researcher triangulation, with both authors collaboratively discussing and finalizing core categories and relationships until consensus was reached. Interviews were conducted by two nursing graduate students who did not participate in subsequent analyses to avoid interpretive bias from participant familiarity.

The first author and participants were colleagues in the same ED and had existing collaborative work. While this “insider” identity facilitated trust‐building and contextual understanding, it risked “familiarity blind spots.” Therefore, interviews were conducted by external graduate students to avoid power dynamics. A “combined insider–outsider” strategy was adopted in data analysis: the first author provided contextualized interpretations, while the second author offered an external critical perspective. For quality control, a three‐member analytical team (including a third‐party researcher) met regularly to challenge interpretations. The first author engaged in reflective journaling to document emotional responses and presuppositions, ensuring that these were explicitly addressed.

The analytic process included three steps:

1. Open coding: Interview transcripts were analyzed line by line using the “breaking” technique. Through persistent questioning (who, when, where, why, how), phenomena were excavated, and data were conceptualized and clustered into categories, with attributes and dimensions clarified.
2. Axial coding: The paradigm model established logical relationships between categories, identifying causal conditions (situational factors), phenomena (core action/interaction categories), context (specific background), intervening conditions (structural factors), action/interaction strategies (approaches to address phenomena), and consequences (outcomes).
3. Selective coding: The core category was identified, a plotline was constructed to integrate all categories, and theoretical sampling was used to verify intercategory relationships until theoretical saturation was achieved.

A dual‐coder independent coding strategy was implemented, with discrepancies resolved through group discussion. The constant comparative method compared new data with existing categories to iteratively refine them. Coding memos documented analytic decisions and theory construction, supporting auditability and methodological rigor.

### 2.6. Rigor and Trustworthiness

This study employed established criteria for ensuring rigor in qualitative research as proposed by Lincoln and Guba [23]: credibility, dependability, confirmability, and transferability.

Credibility refers to the confidence that can be placed in the truth of the findings. Multiple strategies were employed to ensure that the findings accurately represented participants’ experiences. Prolonged engagement: the first author worked in the same ED for 3 years, which fostered deep contextual understanding and trust‐building with participants. Triangulation included (1) researcher triangulation (the first and second authors independently reviewed the coding schemes and challenged interpretations); (2) data source triangulation (participants represented diverse experience levels (1–18 years), professional ranks (nurse to nurse‐in‐charge), education levels (junior college to master’s), and incident types); and (3) methodological triangulation (interviews, field notes, and analytical memos provided multiple data sources). Member checking: the preliminary category system and theoretical model were returned to five participants for confirmation and then refined based on feedback. Peer debriefing: regular meetings with a three‐member analytical team (including a third‐party researcher external to the study) challenged interpretations and identified alternative explanations.

Dependability concerns the consistency and auditability of the research process. An audit trail documented all analytical decisions throughout the process, including interview guide revisions with rationales for each adjustment; coding standards development with exemplar quotations; category modification rationales showing the evolution from initial to final categories; theoretical sampling decisions linking emerging categories to participant selection; and analytical memo documentation recording interpretive processes. A quality control officer maintained these records, enabling the external examination of the research process.

Confirmability addresses the extent to which findings are shaped by participants rather than by researcher bias. The first author maintained reflective journals documenting emotional responses, presuppositions, and potential biases arising from insider status. The “combined insider–outsider” strategy balanced perspectives: the first author provided contextualized interpretations grounded in the ED experience, while the second author offered an external critical perspective without a clinical background. Interviewers were external graduate students without ED experience or prior relationships with participants, avoiding power dynamics that might influence disclosure. Procedural neutrality was maintained through systematic coding procedures, with the second author independently reviewing to minimize subjective arbitrariness.

Transferability pertains to the applicability of findings to other contexts. A thick description of the ED context—including patient volume, staffing patterns, shift structures, and incident reporting procedures—enables readers to assess the similarity between the study setting and their own. Participant characteristics are detailed in Table 1, and incident types are specified, supporting the judgment of applicability. The theoretical model’s abstract nature, focusing on process mechanisms rather than specific content, supports transfer across emergency care contexts. However, cultural adaptation may be necessary, particularly regarding hierarchical authority structures and face‐saving concerns identified in the Chinese context.

## 3. Results

### 3.1. Core Category: Traumatic Recovery and Career Transition

The core category “traumatic recovery and career transition as second victims for emergency nurses” emerged as the central phenomenon explaining how emergency nurses recover from patient safety incidents and achieve professional growth. This category exhibits high generality, explaining intrinsic connections among all main categories and addressing how emergency nurses recover and achieve career transitions.

### 3.2. Theoretical Model Overview

**FIGURE 1:**
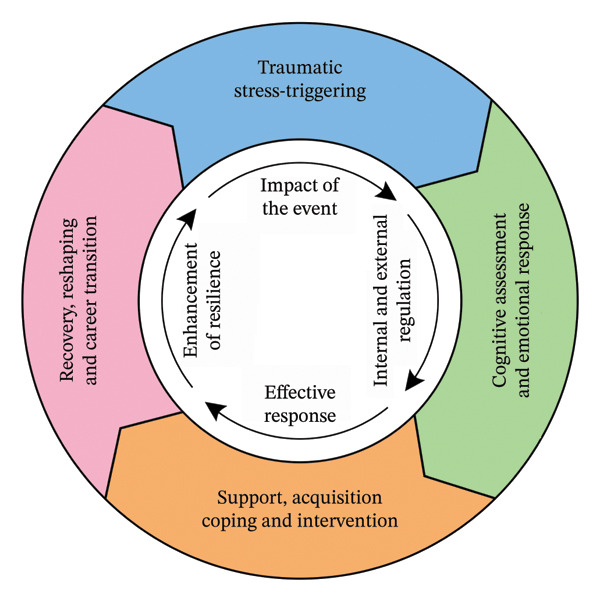
Four‐stage cyclical dynamic model of trauma recovery and career transition.

The analysis yielded 68 initial categories, 22 subcategories, and 7 core categories. These findings informed the construction of a circular, dynamic theoretical model of trauma recovery comprising four sequential stages: trauma stress trigger, cognitive appraisal and emotional response, acquisition of intervention and support, and recovery reconstruction and career reorientation (Figure 1). This model exhibits three key characteristics: (1) procedural (a four‐stage nonlinear evolution with overlapping and recursive features); (2) conditional (context determines strategy selection); and (3) strategic (nurses as active agents who influence outcomes). Within this model, managerial support and proactive coping strategies were identified as important facilitators of trauma recovery.

Theoretical saturation was confirmed through four rounds of theoretical sampling and continuous comparison, with no new attributes, dimensions, or relationships emerging. Table 2 summarizes the core characteristics of each recovery stage and provides illustrative quotations from participants, offering a concise overview of the model’s empirical grounding.

**TABLE 2:** Summary of stage characteristics and illustrative quotations.

Recovery phase	Core characteristics	Illustrative quotation
Traumatic stress‐triggering	Immediate physiological arousal; event suddenness determines intensity	“The instant I realized it, I felt a chill run down my spine” (N1)
Cognitive appraisal and emotional response	Self‐blame; intrusive imagery; professional doubt	“Closing my eyes would immediately bring back vivid images” (N4)
Intervention, coping, and support acquisition	Help‐seeking behaviors; decisive managerial response	“The head nurse first guided me to the waiting room to calm my emotions” (N6)
Recovery reshaping and career transition	Meaning‐making; professional growth; career transition	“I am currently conducting systematic research on nursing management theories” (N18)

### 3.3. Main Categories and Subcategories

Figure 1 illustrates the four‐stage cyclical dynamic model and its constituent categories, while Table 2 summarizes stage‐specific characteristics and participant quotations. The following sections present each stage with its subcategories and supporting evidence from participant narratives.

#### 3.3.1. Traumatic Stress‐Triggering Phase

This phase encompasses nurses’ immediate psychological and physiological responses to patient safety incidents. The type, severity, and suddenness of patient safety events determine psychological reaction intensity. Three subcategories constitute this phase: event characteristics, immediate stress activation, and contextual amplification.

Event characteristics. Participants consistently identified event suddenness and severity as primary determinants of initial trauma intensity. A medication dosage error described by N1 illustrates this: “The instant I realized it, I felt a chill run down my spine.” Similarly, N2 recalled witnessing a patient fall: “The sense of helplessness in watching without being able to intervene still makes my heart race.”

Immediate stress activation. Nurses experienced acute physiological arousal including tachycardia, sweating, and trembling, often accompanied by cognitive narrowing. N3 described “My mind went blank, hands shaking so badly I couldn’t hold the pen to write the incident report.”

Contextual amplification. The ED’s high‐paced environment intensified initial trauma. Time compression eliminated the psychological buffers available in lower acuity settings. N4 noted “In the ED, you don’t have time to process; the next patient is already at the door. The pressure piles up before you can breathe.”

#### 3.3.2. Cognitive Appraisal and Emotional Response Phase

Following the incident, nurses rapidly experienced negative emotional responses, including self‐blame, anxiety, and fear, which may be accompanied by somatic symptoms. At the same time, attribution bias may emerge, leading nurses to question their professional competence and exaggerate perceived occupational risks. Three subcategories define this phase: negative emotional experience, cognitive appraisal bias, and emotional generalization.

Negative emotional experience. Self‐blame dominated participants’ emotional landscape, ranging from transient guilt to persistent self‐denigration. N4 described intrusive imagery: “Closing my eyes would immediately bring back vivid images… I repeatedly doubted whether I was truly suited for this ED role.” N3 reported chronic tension: “I subconsciously attributed it to my own negligence, remaining in a state of chronic mental tension.”

Cognitive appraisal bias. Nurses exhibited systematic distortions in self‐evaluation, disproportionately attributing incidents to personal failure while underestimating systemic factors. N5 explained “I kept thinking if only I had checked one more time, this wouldn’t have happened. I stopped trusting my own judgment.”

Emotional generalization. Trauma responses extended beyond the specific incident to encompass global professional anxiety. N6 described “After the extravasation incident, I became paranoid about every IV line. I was seeing disasters everywhere, even with routine procedures.”

#### 3.3.3. Intervention, Coping, and Support Acquisition Phase

Nurses engaged in coping behaviors either proactively or reactively and sought support from multiple sources. A nonblaming managerial attitude and timely, proactive support are critical in alleviating psychological distress and facilitating trauma recovery. Two main categories constitute this phase: perceived social support and strategic response options.

Perceived social support. Support sources included colleagues, managers, family, and professional psychological services. Managerial response emerged as decisive. N6 contrasted two experiences: “The head nurse first guided me to the waiting room to calm my emotions… This professional empathy significantly alleviated my psychological burden.” Conversely, N7 recounted “When the head nurse said ‘this is your responsibility’ in front of everyone, I wanted to disappear. I never reported another incident after that.”

Colleague support ranged from nonverbal companionship to active listening and guidance. N8 valued peer understanding: “Senior nurses who’d been through similar events—just their presence, no words needed—made me feel less alone.”

Strategic response options. Nurses adopted passive avoidance, active problem‐solving, or professional intervention. N8 demonstrated active coping: “I proactively contacted the psychology department, learned to view this adverse event rationally.” N9 initially chose avoidance: “I called in sick for 3 days, couldn’t face the unit. But hiding made it worse—the guilt grew every day.”

#### 3.3.4. Recovery Reshaping and Career Transition Phase

Nurses gradually accepted the incident, reconstructed cognitive meaning, and achieved psychological recovery. Traumatic experiences transformed into motivation for professional growth, resulting in optimized clinical practice or adjusted career development trajectories. Two subcategories define this phase: trauma recovery process and professional transition and development.

Trauma recovery process. Recovery proceeded through acute stress response, adaptation, and psychological restructuring. N10 described meaning‐making: “I now systematically share this case to emphasize… the dual value of safeguarding patient safety and mitigating occupational risks.”

Professional transition and development. Successful recovery often catalyzed career advancement. Three trajectories emerged (1) clinical practice optimization (N11 adopted stricter verification protocols), (2) transition to nursing management (N12 pursued head nurse training), and (3) academic/educational pathways (N18 reported “I am currently conducting systematic research on nursing management theories, with the aim of developing targeted support programs.”)

N13 summarized the transformative potential: “That incident broke me but also rebuilt me. I’m a better nurse now—more careful, more compassionate, and strangely more confident.”

## 4. Discussion

This study developed a four‐stage cyclical model of trauma recovery among emergency nurses as second victims, comprising (1) traumatic stress trigger, (2) cognitive appraisal and emotional response, (3) intervention and support acquisition, and (4) recovery reconstruction and career reorientation. The model revealed that managerial support appeared to facilitate nurses’ progression from psychological recovery to professional growth. Unlike linear recovery frameworks, this model demonstrated overlapping phases, potential regression, and upward‐spiraling trajectories, where successful recovery enhances resilience for future events. These findings challenge the conventional conceptualization of second victim experiences as purely psychological injuries, instead positioning them as potential catalysts for professional development when appropriate support systems are in place. The following sections elaborate on the theoretical significance of this model, central role of managerial support, transformative value of traumatic experiences, and practical implications for emergency nursing management.

The four‐stage cyclical dynamic trauma recovery model offers distinctive theoretical contributions compared with existing frameworks. Unlike Scott et al.’s three‐phase model (acknowledge−cope−recover) [24] and Burlison et al.’s seven‐phase trajectory [25], this study revealed how emergency settings’ “high‐pressure−high‐frequency−high‐mobility” features fundamentally reshape recovery mechanisms. Emergency nurses exhibit temporal compression—immediate acute stress without the psychological buffers available in ward settings—and role ambiguity stemming from team‐based responsibility attribution, differing qualitatively from ward nurses’ more linear recovery patterns [26]. While existing models assume “returning to baseline,” this study found that emergency nurses’ recovery involves permanent professional identity reconstruction rather than mere restoration. The model also breaks the linear “stress‐response” framework by integrating psychological resilience [27] and post‐traumatic growth theories [28], proposing a spiral mechanism: stress triggering ⟶ recovery transformation ⟶ resilience enhancement ⟶ optimized coping. This aligns with the psychological safety theory [29], where managerial support serves as a critical mediating condition for a “blame‐free culture.” However, this model reveals the stage‐specific nature of such culture: nonblaming attitudes during crises and career empowerment during recovery operate through distinct mechanisms, expanding the psychological safety theory’s trauma application. Furthermore, by positioning career transformation as a core outcome, the model surpasses prior studies limited to psychological rehabilitation [30]. Engaging the “just culture” theory [31], it demonstrates that reframing incidents as learning opportunities rather than accountability events transforms trauma into “career catalysts,” translating the macrolevel concept into actionable phased interventions.

The four stages exhibit phasic dynamic characteristics, showing overlapping, recursive, and upward‐spiraling features rather than linear progression. Overlap occurs when nurses simultaneously experience “coping phase”‐related help‐seeking behaviors during the “cognitive appraisal phase,” blurring stage boundaries. Recursiveness manifests when insufficient support causes regression from the “coping phase” back to the “emotional response phase,” forming a retreat trajectory. Spiraling upward describes how nurses completing the cycle successfully demonstrate stronger resilience and more effective coping in future incidents, validating the “resilience enhancement ⟶ optimized coping” mechanism. Managerial interventions, thus, require phase sensitivity: a nonblaming attitude during crises may serve as the cycle’s entry condition, while professional empowerment during recovery reinforces the upward‐spiraling trajectory.

Drawing on extensive interview data, this study demonstrated that managerial support is the most influential external factor shaping trauma recovery outcomes for emergency nurses as second victims, consistent with prior research [32–35]. Participants consistently reported that the head nurse’s response played a decisive role in their recovery trajectory. Nonblaming attitudes and proactive support effectively alleviated self‐blame, reduced psychological distress, and restored professional confidence. Conversely, blame‐oriented criticism intensified psychological trauma and contributed to incident concealment and avoidance behaviors. These findings emphasize the need for nursing managers to move from a traditional “blame culture” to a “fairness culture” philosophy [36], reframing incident management from individual fault attribution to systemic process involvement. Nursing managers should receive structured training in secondary victim support strategies to enhance their capacity to recognize psychological distress and provide timely, appropriate support. This will enable them to serve as a stable, credible support system for emergency nurses navigating occupational trauma.

An important finding is that career development following trauma recovery constitutes a meaningful outcome of secondary victim experiences. Nurses who successfully progressed through recovery did not remain constrained by psychological distress; rather, many reported strengthened professional identity and improved clinical practice, such as stricter adherence to verification procedures and heightened sensitivity to changes in patient conditions. Furthermore, some nurses described deliberate transitions into nursing management or education roles, transforming their traumatic experiences into resources for team learning and organizational development. These findings challenge the conventional assumption that “trauma equals occupational injury” and highlight the potential developmental value embedded in traumatic experiences [17, 37]. From a nursing management perspective, this emphasizes the importance of recognizing and harnessing post‐recovery growth. Providing targeted professional development opportunities—such as involvement in patient safety initiatives, mentoring junior nurses, or participating in emergency response planning—may enable nurses to translate recovery into sustained professional advancement, fostering a virtuous cycle linking individual growth, team capacity building, and patient safety enhancement.

Based on the cyclical dynamic model, managers should design a phased, continuous, and iterative support system that accounts for overlaps and regressions between phases. Nurses may return to the “emotional response phase” owing to insufficient support during the “coping phase” or re‐enter the “stress phase” triggered by new events during “recovery.” Intervention strategies must maintain flexibility through three mechanisms. First, phase overlap management: nurses may experience multiple phases simultaneously (e.g., psychological counseling while considering job transfers); hence, it is necessary to avoid rigid segmentation and provide integrated support packages combining psychological and career development assistance. Second, recursive risk prevention: it is important to identify regression triggers such as new events during recovery or sudden discontinuation of support and establish dynamic monitoring mechanisms allowing nurses to “step back” to previous phases to address unresolved emotions. Third, spiral reinforcement mechanism: it is crucial that each successful cycle contributes to organizational capital, and after recovery, nurses can serve as peer supporters, creating a virtuous cycle of “assisted ⟶ self‐help ⟶ assisting,” thereby enhancing the team’s overall resilience.

Practical strategies based on the recovery stage include four components. During the traumatic stress‐triggering phase, immediate crisis intervention should be provided: a quiet, private space for emotional stabilization paired with a psychologically resilient senior nurse [20], temporary reassignment from high‐risk duties to reduce acute stress exposure, and avoidance of evaluative criticism until systematic investigation is complete to minimize secondary psychological harm. During the cognitive assessment and emotional response phase, targeted psychological support should be offered: professional one‐on‐one counseling including cognitive behavioral therapy to address maladaptive self‐attribution and reduce negative emotions [38], structured peer support groups for emotional expression and shared experience, and regular resilience‐focused training to enhance psychological adjustment capacity. During the intervention response and support acquisition phase, multidimensional support should be given: a three‐tier support framework comprising the head nurse, Departmental Psychological Specialist, and Hospital Psychology Department; nonblaming incident management procedures that emphasize systemic weaknesses over individual faults; and unobstructed access to psychological services with simplified referral pathways for timely intervention. During the recovery and reformation stage, career development empowerment should be provided: targeted opportunities including management role exposure, faculty development training, and research skill enhancement; invitation to contribute to patient safety system development, transforming individual experiences into collective resources; and dissemination of exemplary post‐traumatic growth cases to cultivate a supportive culture recognizing trauma while encouraging proactive professional growth.

Application of this model in the Chinese context requires specific adjustments based on organizational analysis and contextualization. Staffing differentiation necessitates additional security assurances for contract nurses, such as explicit commitments that incidents will not directly affect contract renewals. Face management requires establishing anonymous platforms for sharing experiences to transform individual narratives into collective knowledge without exposing identities. Administrative empowerment should incorporate head nurse training into the cadre evaluation system to enhance institutional incentives for supportive behaviors. Gradual return to work in high‐workload scenarios should adopt a “shadowing buddy” model—pairing recovering nurses with highly resilient nurses—rather than completely suspending work.

This study has several limitations. First, contextual specificity: the single‐center sample from Chinese tertiary EDs needs validation in primary care or other medical cultures. The prevalent “hierarchical authority culture” in Chinese hospitals may amplify head nurses’ decisive role, which could manifest differently in flat management cultures. Regarding power dynamics, graduate student interviews may reduce defensiveness but overlook deeper concerns of senior nurses; the sample lacked associate chief nurses or higher ranking professionals. Second, demographic representation: the female‐dominated sample (16:2) reflects general nursing demographics; however, male nurses may face unique “masculine gender expectations” and help‐seeking barriers, warranting dedicated research. Third, theoretical saturation: although four rounds were conducted, recovery trajectories for specific incident types (e.g., workplace violence) may differ, indicating that further theoretical saturation is necessary for specialized populations.

## 5. Conclusions

This study employed Strauss and Corbin’s grounded theory to explore trauma recovery pathways of emergency nurses as second victims, constructing a theoretical model of “trauma recovery and professional transformation.” Central to this model is the finding that, within emergency care contexts, managerial support was identified as a key factor facilitating nurses’ progression from “psychological recovery” to “professional growth” through phased interventions, thereby forming an upward‐spiraling dynamic mechanism.

These findings contribute to theory development by integrating trauma psychology, organizational behavior, and nursing management, proposing a context‐specific recovery theory that transcends linear frameworks and singular psychological rehabilitation perspectives prevalent in existing research. From a practical standpoint, the model equips nursing administrators with phased, precise, and developmental support strategies that translate the “just culture” concept into actionable clinical practices. Ultimately, this achieves a triple enhancement of nurses’ well‐being, team learning, and patient safety.

Looking ahead, several research directions deserve attention. First, cross‐cultural validation is needed to compare the model’s applicability across collectivist and individualist contexts. Second, quantitative validation could be achieved by developing an “Emergency Nurse Second Victim Recovery Scale” to test stage divisions and cyclical mechanisms. Third, longitudinal tracking employing prospective designs would help verify temporal sequences of cyclical dynamics. Finally, intervention experiments should design phased support programs based on the model and conduct randomized controlled trials.

## Funding

This research received no specific grant from any funding agency in the public, commercial, or not‐for‐profit sectors.

## Disclosure

The work was carried out as part of the authors’ institutional employment.

## Conflicts of Interest

The authors declare no conflicts of interest.

## Data Availability

The data are not publicly available owing to privacy or ethical restrictions. However, deidentified excerpts from interviews or transcripts can be provided upon reasonable request.
